# TWEAKing the Hippocampus: The Effects of TWEAK on the Genomic Fabric of the Hippocampus in a Neuropsychiatric Lupus Mouse Model

**DOI:** 10.3390/genes12081172

**Published:** 2021-07-29

**Authors:** Dumitru A. Iacobas, Jing Wen, Sanda Iacobas, Chaim Putterman, Noa Schwartz

**Affiliations:** 1 Personalized Genomics Laboratory, Center for Computational Systems Biology, Roy G Perry College of Engineering, Prairie View A&M University, Prairie View, TX 77446, USA; daiacobas@pvamu.edu; 2 Dominick P. Purpura Department of Neuroscience, Albert Einstein College of Medicine, Bronx, NY 10461, USA; 3 Department of Medicine (Rheumatology), Albert Einstein College of Medicine, Bronx, NY 10461, USA; jing.wen1@rutgers.edu (J.W.); chaim.putterman@einsteinmed.org (C.P.); 4 Department of Pathology, New York Medical College, Valhalla, NY 10595, USA; sandaiacobas@gmail.com; 5 Department of Microbiology & Immunology, Albert Einstein College of Medicine, Bronx, NY 10461, USA; 6 Azrieli Faculty of Medicine, Bar-Ilan University, Zefat 1311502, Israel; 7 Galilee Medical Center Research Institute, Nahariya 22100, Israel

**Keywords:** neuropsychiatric lupus, hippocampus, TNF-like weak inducer of apoptosis (TWEAK), Tnfsf12, Fn14, Tnfrsf12a, Akt2, PI3K-AKT pathway, Dnajc28, Syne2, transthyretin

## Abstract

Neuropsychiatric manifestations of systemic lupus erythematosus (SLE), specifically cognitive dysfunction and mood disorders, are widely prevalent in SLE patients, and yet poorly understood. TNF-like weak inducer of apoptosis (TWEAK) has previously been implicated in the pathogenesis of neuropsychiatric lupus (NPSLE), and we have recently shown its effects on the transcriptome of the cortex of the lupus-prone mice model MRL/lpr. As the hippocampus is thought to be an important focus of NPSLE processes, we explored the TWEAK-induced transcriptional changes that occur in the hippocampus, and isolated several genes (*Dnajc28*, *Syne2*, *transthyretin*) and pathways (PI3K-AKT, as well as chemokine-signaling and neurotransmission pathways) that are most differentially affected by TWEAK activation. While the functional roles of these genes and pathways within NPSLE need to be further investigated, an interesting link between neuroinflammation and neurodegeneration appears to emerge, which may prove to be a promising novel direction in NPSLE research.

## 1. Introduction

Neuropsychiatric Lupus (NPSLE) is one of the most prevalent manifestations of Sys-temic Lupus Erythematosus (SLE), occurring in up to 80% of lupus patients [1]. The 1999 American College of Rheumatology (ACR) ad hoc committee defined 19 clinical syndromes as manifestation of NPSLE, and those range from acute, overtly inflammatory presentations such as psychosis, transverse myelitis, and chorea to more subtle, non-specific symptoms such as headaches, mood disorders, and cognitive dysfunction [2]. Naturally, the more nebulous manifestations are significantly more prevalent (cognitive dysfunction and mood disorders range from 6.6% to 80%, while acute confusional state and cranial neuropathy affect 0.9–7% of patients [1]), but are more difficult to attribute directly to SLE, partly because they commonly occur regardless of systemic disease activity. Due to their apparent non-inflammatory presentation, cognitive dysfunction and mood disorders are thought to often be related to secondary causes, such as the patients being in a chronic disease state, neuro-affective medication use, or structural brain damage due to cerebrovascular disease, among others. Still, the fact that several lupus mice models pre-sent with similar neuropsychiatric clinical features [1]; advanced imaging studies of SLE patients demonstrate changes in neural networks that are common to patients compared with healthy controls [3]; in many patients, NPSLE manifestations occur early in the disease process, prior to the onset of chronic complications or treatment initiation [4,5], strongly support the notion that there are primary disease-related processes that lead to neurocognitive and psychiatric dysfunction in SLE. The non-specific nature of many of the NPSLE symptoms, and our current lack of understanding of the underlying processes make the diagnosis challenging, and directed treatment options scarce [6,7].

MRL/MpJ-Fas^lpr/lpr^ (MRL/lpr) lupus prone mice have a loss-of-function mutation in the *Fas* gene, superimposed on a complex MRL background. These mice manifest a systemic lupus-like phenotype, including anti-nuclear antibody formation, immune-complex mediated glomerular disease, and typical skin manifestations [8], as well as a range of cognitive and affective symptoms, with memory deficits and depression-like behavior [9]. Their neuropsychiatric behavior is evident as early as 8 weeks of age and peaks at a median of 16 weeks of age [10]. This protracted disease course together with systemic and neurologic presentation that is similar to human disease make this a popular model for NPSLE.

Tumor necrosis factor (TNF)-like weak inducer of apoptosis (TWEAK or TNFSF12), and its cognate receptor, Fn14 (TNFRSF12A), have been shown to play an important role in SLE in general [11,12,13] and NPSLE in particular. TWEAK, a secreted member of the TNF-ligand superfamily, and Fn14 are expressed in astrocytes, microglia, brain microvascular endothelial cells, and neurons; and their interaction activates pro-inflammatory cytokine production, among other effects [14,15,16]. MRL/lpr Fn14 knock-out mice (Fn14ko) display less depression and neurocognitive dysfunction than their background controls [17], and administration of TWEAK to the cerebrospinal fluid (CSF) of non-lupus mice induces neurocognitive behavioral changes [18]. Furthermore, NPSLE was associated with high TWEAK levels in the CSF of patients [19].

In a recent manuscript, we analyzed and compared the transcriptome of the cortex of the MRL/lpr lupus mouse model to the cortices of Fn14ko and non-lupus, MRL/MpJ (MRL/+), background control [20]. Utilizing a novel analysis method, incorporating both expression levels of the specific genes with their overall genetic importance to the model’s transcriptome, we identified several pathways that appear to have significant impact on the models’ differential phenotype. The MRL/lpr model displayed significant changes in neurotransmission processes, particularly in the dopaminergic pathway compared with Fn14ko and background MRL/+ controls. In addition, the Phosphoinositide 3-kinase (PI3K)-AKT intracellular pathway was identified as playing a role in the TWEAK-Fn14-mediated effects on NPSLE in the MRL/lpr mice. To further examine and validate the importance of these pathways, we proceeded to analyze the transcriptome of the cells in the hippocampus of the MRL/lpr mice compared with Fn14ko and background controls. The hippocampus has for long been implicated as a critical focus of neurocognitive and mood manifestation of NPSLE [21,22,23,24,25,26]. Thus, a careful analysis of its transcriptome may provide more specific and pertinent information regarding critical pathways in NPSLE, thereby identifying potential diagnostic markers, as well as promising treatment targets.

## 2. Materials and Methods

### 2.1. Animals

As described previously [20], MRL/lpr mice and Fn14ko (backcross generation #8) littermates were bred at Biogen Idec (Cambridge, MA, USA) and transferred to Albert Einstein College of Medicine (AECOM) at 8–10 weeks of age. Control MRL/+ mice were purchased from the Jackson Laboratory (Bar Harbor, ME, USA). Housing conditions were controlled, with a temperature of 21–23 °C and a 12:12 h light:dark cycle. All animal study protocols were approved by the institutional animal care and use committee (IACUC) at AECOM (protocol# 20170516).

There were 4 MRL/lpr, 4 Fn14ko and 4 MRL/+ mice used for this study. All mice were female and sacrificed at the diestrus phase of their hormonal cycle. At sacrifice, all were within one week of age (about 12 weeks old), and all were sacrificed within a 2-week time period. Following the sacrifice, the hippocampus was isolated, and immediately processed.

### 2.2. Transcriptomics

We used the experimental protocol and analyses presented in the previous report [20]. The hippocampus of each of the four mice from every group (MRL/+, MRL/lpr, and Fn14ko) was profiled separately. After reversed transcription in the presence of Cy3/Cy5 dUTP, total RNAs with different fluorescent labels from pairs of biological replicas were co-hybridized 17 h overnight at 65 °C with microarrays of 4 × 44 k Agilent 60-mer G2519F mouse chips.

The spots with corrupted pixels or with foreground fluorescence less than twice the background were eliminated from the analysis. Valid background subtracted foreground signals were normalized to the median and the results averaged separately for each group of spots probing redundantly the same gene.

Through the Genomic Fabric Paradigm (GFP) approach [27] we took full advantage of quantifying tens of thousands of genes at a time on four biological replicas. Thus, each quantified gene “*i*” in each region “*B*” (= cortex, hippocampus) and each phenotype “*P*” (= MRL/+, MRL/lpr, Fn14ko) was assigned the independent measures: average expression level (*AVE*), relative expression variability (*REV*) and expression correlation with each other gene (*COR*) according to the definitions (1)–(3):

(1) $$AVE_{i}^{\left(B;P\right)}=\frac{1}{R_{i}}\sum_{k=1}^{R_{i}}\mu_{i,k}^{\left(B;P\right)}=\frac{1}{R_{i}}\sum_{k=1}^{R_{i}}\underset{\mu_{i,k}^{\left(B;P\right)}}{\underbrace{\left(\frac{1}{4}\sum_{\xi =1}^{4}a_{i,k,\xi }^{\left(B;P\right)}\right)}}\;,\;where:$$

B = cortex, hippocampus

$R_{i}$ = number of micro array spots probing redundantly gene *i*

$a_{i,k,\xi }^{\left(B;P\right)}$ = expression of gene “*i*” probed by spot “*k*” on biological replica “ξ”

$a_{i,k,\xi }^{\left(B;P\right)}$ = average expression of gene “*i*” probed by spot “*k*” on all biological replicas

(2) $$REV_{i}^{\left(B;P\right)}=\underset{\mathrm{correction}\;\mathrm{coefficient}}{\underbrace{\frac{1}{2}\left(\sqrt{\frac{r_{i}}{\chi^{2}\left(r_{i};0.975\right)}}+\sqrt{\frac{r_{i}}{\chi^{2}\left(r_{i};0.025\right)}}\right)}}\sqrt{\underset{\mathrm{pooled}\;\mathrm{CV}}{\underbrace{\frac{1}{R_{i}}\sum_{k=1}^{R_{i}}{\left(\frac{s_{ik}^{\left(B;P\right)}}{\mu_{ik}^{\left(B;P\right)}}\right)}^{2}}}}\times 100\%$$

$\chi^{2}$ = chi-square score for $r_{i}$ degrees of freedom and α = 0.05

$s_{ik}$ = standard deviation of the expression of gene “*i*” probed by spot “*k*” on all biologicals replicas

$r_{i}=4R_{i}-1$ = number of degress of freedom

(3) $$COR_{i,j}^{\left(B;P\right)}=\frac{\sum_{k=1}^{R_{i}}\left(\left(\frac{1}{4}\sum_{\xi =1}^{4}a_{i,k,\xi }^{\left(B;P\right)}-\mu_{i,k}^{\left(B;P\right)}\right)\left(\frac{1}{4}\sum_{\xi =1}^{4}a_{j,k,\xi }^{\left(B;P\right)}-\mu_{j,k}^{\left(B;P\right)}\right)\right)}{\sqrt{\sum_{k=1}^{R_{i}}{\left(\frac{1}{4}\sum_{\xi =1}^{4}a_{i,k,\xi }^{\left(B;P\right)}-\mu_{i,k}^{\left(B;P\right)}\right)}^{2}\sum_{k=1}^{R_{i}}{\left(\frac{1}{4}\sum_{\xi =1}^{4}a_{j,k,\xi }^{\left(B;P\right)}-\mu_{j,k}^{\left(B;P\right)}\right)}^{2}}}\;(\mathrm{Pearson}\;\mathrm{correlation}\;\mathrm{cefficient})$$

Relative Expression Variability (*REV*) among biological replicas shows how much cellular homeostatic mechanisms control the transcript abundance against environmental slight random fluctuations [28], and the noise associated with the stochastic nature of the chemical reactions involved in the gene transcription. Genes critical for cell survival, proliferation, and integration in the multicellular structures are under strict control, while control of genes ensuring cell adaptation to environmental fluctuation is much more lenient.

Pearson pair-wise product-momentum correlation coefficient of expression levels (*COR*) with each other gene in that region and phenotype, or correlation with the same gene in other regions, reflects the Principle of Transcriptomic Stoichiometry [29], a generalization of Dalton’s Law of Multiple Proportions [30]. This principle states that genes networked in functional pathways are expressed in definite proportions, even under environmental fluctuations.

By combining *REV* and *COR*, we established gene hierarchy and identified Gene Master Regulator (GMR) in each region and each phenotype using Gene Commanding Height (*GCH*) [31,32,33]:

(4) $$GCH_{i}^{\left(B;P\right)}\equiv \frac{{\langle REV\rangle }^{\left(B;P\right)}}{REV_{i}^{\left(B;P\right)}}\exp\left(\frac{4}{N}{\sum_{j\in ALL,j\neq i}\left(COR_{ij}^{\left(B;P\right)}\right)}^{2}-1\right)$$

We compared AVEs of a gene in two phenotypes/regions and identified statistically significantly regulated/differentially expressed genes using the composite criterion of absolute fold change, exceeding the combined contributions of the expression variabilities, and the *p*-value of the heteroscedastic *t*-test being less than 0.05 (5). The expression ratio x was defined to clearly indicate the extent of the up- (positive ratio) or down- (negative ratio) regulation:

(5) $$\left|x_{i}^{\left(B1;P1\;\mathrm{vs}\;B2;P2\right)}\right|>CUT_{i}^{\left(B1;P1\;\mathrm{vs}\;B2;P2\right)}=1+\frac{1}{100}\sqrt{2\left({\left(REV_{i}^{\left(B1;P1\right)}\right)}^{2}+{\left(REV_{i}^{\left(B2;P2\right)}\right)}^{2}\right)}\;\wedge \;p_{i}^{\left(B1;P1\;\mathrm{vs}\;B2;P2\right)}<0.05$$

where:

$$x_{i}^{\left(B1;P1\;\mathrm{vs}\;B2;P2\right)}\equiv \left\{\begin{array}{cc} \frac{\mu_{i}^{\left(B2;P2\right)}}{\mu_{i}^{\left(B1;P1\right)}} & ,\;\mathrm{if}\;\mu_{i}^{\left(B2;P2\right)}\geq \mu_{i}^{\left(B1;P1\right)}\\ -\frac{\mu_{i}^{\left(B1;P1\right)}}{\mu_{i}^{\left(B2;P2\right)}} & ,\;\mathrm{if}\;\mu_{i}^{\left(B2;P2\right)}<\mu_{i}^{\left(B1;P1\right)} \end{array}\right.\;\mathrm{expression}\;\mathrm{ratio}\;(\mathrm{negative}\;\mathrm{for}\;\mathrm{down}-\mathrm{regulation})\;$$

Traditionally, transcriptomic alterations are quantified by the percentages of up- and down-regulated genes. Not only is this method limited to only significantly regulated genes but it considers each affected gene as an equal +1 or −1 contributor.

For a more comprehensive characterization of the contribution of individual genes and functional pathways “Γ” to the expression difference between the compared phenotypes, we computed the Weighted Individual (Gene) Regulation (*WIR*) and the Weighted Pathway Regulation (*WPR*):

(6) $$\begin{array}{l} WIR_{i}^{\left(P1\;vs\;P2\right)}\equiv AVE_{i}^{\left(P2\right)}\underset{\mathrm{regulation}\;\mathrm{sign}}{\underbrace{\frac{x_{i}^{\left(P1\;vs\;P2\right)}}{\left|x_{i}^{\left(P1\;vs\;P2\right)}\right|}}}\underset{\mathrm{absolute}\;\mathrm{net}\;\mathrm{fold}-\mathrm{change}}{\underbrace{\left(\left|x_{i}^{\left(P1\;vs\;P2\right)}\right|-1\right)}}\underset{\mathrm{confidence}\;\mathrm{of}\;\mathrm{the}\;\mathrm{regulation}}{\underbrace{\left(1-p_{i}^{\left(P1\;vs\;P2\right)}\right)}}\\ WPR_{\Gamma }^{\left(P1\;vs\;P2\right)}\equiv \sqrt{\frac{1}{Card(\Gamma )}\sum_{i=1}^{Card(\Gamma )}{\left(WIR_{i\in \Gamma }^{\left(P1\;vs\;P2\right)}\right)}^{2}}\; \end{array}$$

where:

P1=MRL/lpr, Fn14ko ∧P2= MRL/+, Fn14ko ∧P2≠P1Card(Γ) = number of quantified genes in the partway Γ

As shown above, beyond the net-fold change, *WIR* takes into account the reference expression level of the gene and the statistical confidence (1 − *p*-value) of its regulation.

## 3. Results

Raw and normalized gene expression data were deposited and are publicly accessible at https://www.ncbi.nlm.nih.gov/geo/, (accessed on 25 March 2021) as GSE164140 (cortex) and GSE169486 (hippocampus). In total, we quantified 16,863 unigenes in each of all 24 profiled samples (2 regions × 3 phenotypes × 4 biological replicas). GFP approach turned expression data into: 101,178 average expression levels (AVE), 101,178 relative expression variabilities (REV), and 853,031,718 expression correlations (COR) among distinct genes in the same region and phenotype, and 50,679 between-regions correlations of the same genes. Thus, by fully exploiting the transcriptomic profiles, the workable experimental data was increased by 16,945 times of what is traditionally used in gene expression studies limited to only the average expression levels. 

### 3.1. Independent Expression Characteristics of Individual Genes

**Figure 1:**
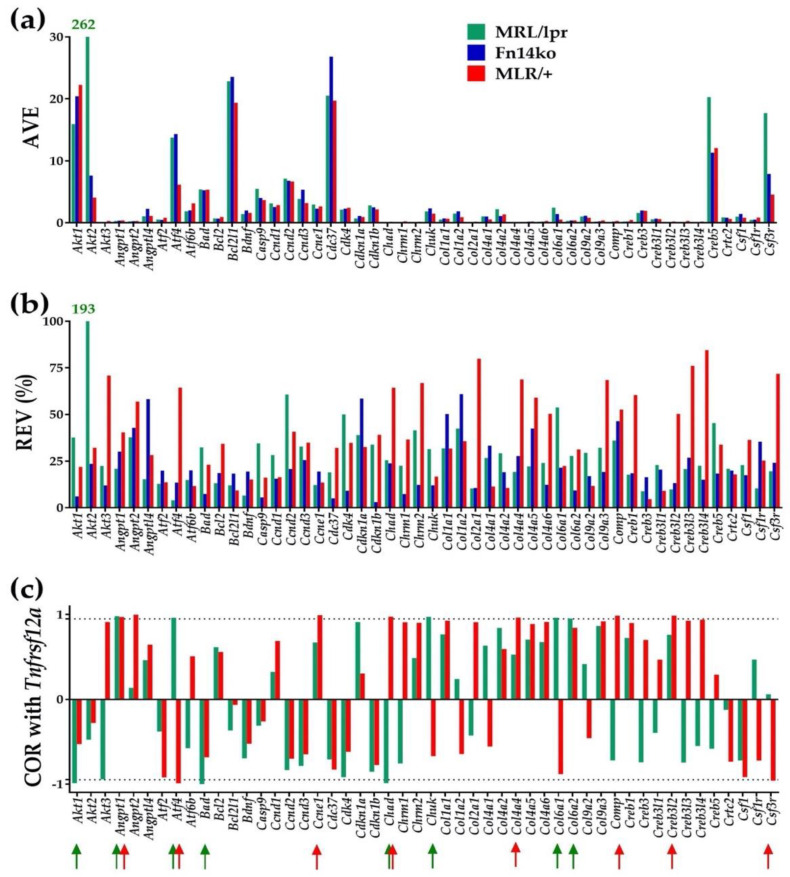
The independent characteristics of the first 50 alphabetically ordered hippocampal PI3K-AKT genes in the three phenotypes. (**a**) Average expression level (AVE); (**b**) Relative expression variability (REV); (**c**) Pair-wise product-momentum correlation coefficient (COR) of PI3K-AKT genes with *Tnfrsf12a.* Red/green arrows indicate the statistically significant (*p* < 0.05) correlations in the MRL/lpr and MRL/+ mice. With AVE = 262 and REV = 193, *Akt2* is the most remarkable gene of the selection in the hippocampus of the MRL/lpr mice.

For illustrative purposes, Figure 1 presents the average expression level (AVE), relative expression variability (REV) and correlation coefficient (COR) with *Tnfrsf12a* (*Fn14*) of the first 50 alphabetically ordered genes involved in the hippocampal PI3K-AKT signaling pathway of MRL/lpr, Fn14ko and MRL/+ mice. AVE indicates the expression level of each gene, and REV examines the genes’ degree of variability within each mouse model. It is assumed that genes that are critical for cell survival and function are highly preserved (low REV), and those that are meant to allow for adaptation would display higher REV. COR indicates the correlation of each gene’s expression with *Tnfrsf12a* expression. As Fn14ko mice do not express *Tnfrsf12a*, this model was not included in the COR analysis. Of note is the obvious independence of these three characteristics within each phenotype and the differences among the three models. Table 1 presents the 20 most expressed genes (highest AVE) in the hippocampus of each phenotype. Supplementary Tables S1 and S2 present the 20 most stably (low REV) and unstably (high REV) expressed genes in each phenotype and the corresponding values in the other two phenotypes. 

As shown in Figure 1a,b, as well as Table 1, and in line with our previous cortex evaluation [20], *Akt2* is highly expressed in the hippocampus of the MRL/lpr mice, and shows substantially increased REV in the MRL/lpr mice compared with both Fn14ko and MRL/+ controls. The apparent normalization of *Akt2* expression in the Fn14ko model potentially points to *Akt2* being directly related to TWEAK/Fn14 pathway activation. As discussed previously, the lack of correlation of *Akt2* with *Tnfrsf12a* in the lupus mouse model (Figure 1c) may be due to a discrepancy between *Tnfrsf12a* gene expression (quantified here) and its activation.

Table 1 identifies *Dnajc28* (DnaJ heat shock protein family 40 member C28) as the gene with the largest AVE in MRL/lpr compared with both Fn14ko and MRL/+. *Dnajc28* was previously implicated in the pathogenesis of neurodegenerative diseases, such as Alzheimer’s Disease and Parkinson’s Disease [34,35,36], possibly as a protective protein that is expressed in high level in the setting of local injury or toxicity [37]. Its high level of expression in this case, therefore, is likely a compensatory mechanism to the TWEAK/Fn14-induced inflammation and its subsequent local damage.

### 3.2. Measures of Expression Regulation

**Figure 2:**
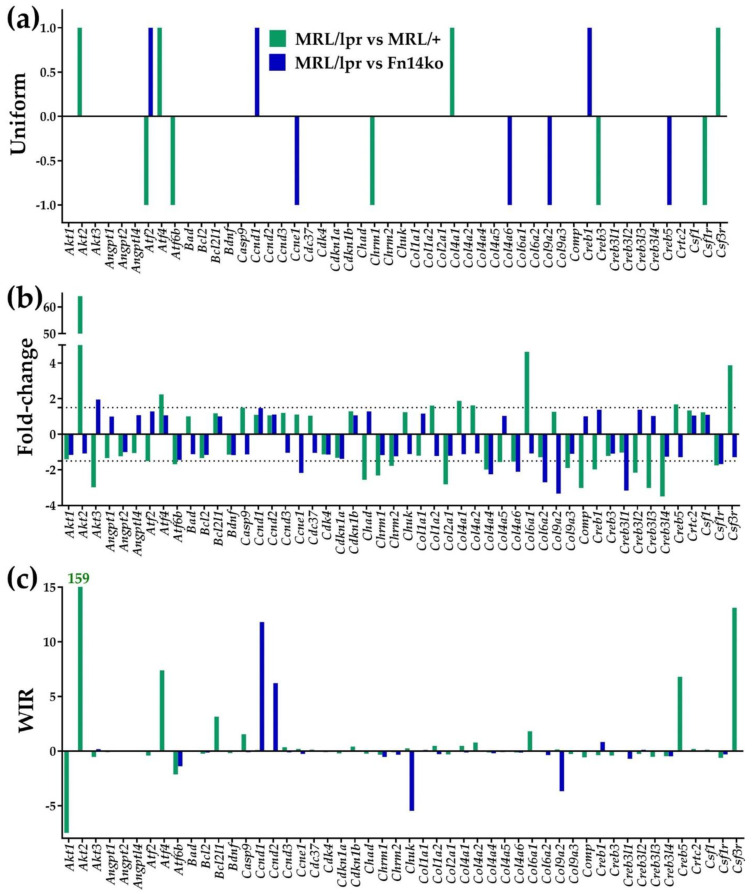
Measures of differential expression of the first 50 alphabetically ordered hippocampal PI3K-AKT genes in the MRL/lpr mouse with respect to the two controls. (**a**) Uniform contribution (+1 or −1) of the significantly regulated genes. (**b**) Fold-change (negative for down-regulation) of all genes within the selection. (**c**) Weighted Individual (gene) Regulation (WIR). In addition to the net fold-change, WIR considers also the reference expression level of the gene and the statistical confidence (1 − *p*-value) of its regulation. Taken together, it is a comprehensive measure of the relative contribution of the gene to the model’s transcriptome.

Figure 2 presents the uniform contribution (reported as +1/−1, reflecting statistically significant up/down-regulated genes), expression ratio (or “fold-change”, negative for down-regulation) and weighted individual gene regulation (WIR) for the first 50 alphabetically ordered PI3K-AKT genes. The figure emphasizes the additive effect provided by the expression ratio (Figure 2b) and WIR (Figure 2c) measures, compared to the traditional uniform contribution analysis (Figure 2a). As discussed previously, the uniform contribution only identifies the genes that are significantly up-/down-regulated, without quantifying the level of regulation or its impact on the model’s transcriptome. Expression ratio discriminates the genes with respect to the magnitude of their regulation, while WIR (Figure 2c) weighs the net fold-change by the reference expression level and the statistical confidence of the regulation, thus providing a more comprehensive measure of transcriptomic impact. 

Table 2 lists the 60 highest-contributor genes to the MRL/lpr model, compared with Fn14ko and MRL/+ controls, based on expression ratio and WIR values.

As demonstrated in Figure 2c and Table 2, *Akt2* is among the most impactful genes within the PI3K-AKT pathway by an order of magnitude compared with other genes’ WIR. At the same time, as shown in Table 2, other genes also seem to play significant roles in regulating the genomic fabric of the different mice phenotypes through TWEAK/Fn14 activation (highlighted genes in Table 2 show similar effect of up- or down-regulation in both Fn14ko and MRL/+ controls compared with MRL/lpr indicating a likely role for the TWEAK/Fn14 pathway in these gene’s regulation). Of those, the most notable are *Dnajc28*, *Syne2* (synaptic nuclear envelope 2) that are upregulated in the MRL/lpr model compared with both Fn14ko and MRL/+, as well as transthyretin (*Ttr*) that is downregulated in the lupus-prone mice compared with both controls. All three of these genes have been previously implicated in either CNS pathology [34,35,38,39,40,41,42] or autoimmunity [43], making them interesting targets of further study within the context of NPSLE. 

### 3.3. Regulation of the Genomic Fabrics Responsible for Neurotransmission, Chemokine Signaling and PI3K-AKT Signaling Pathways

**Figure 3:**
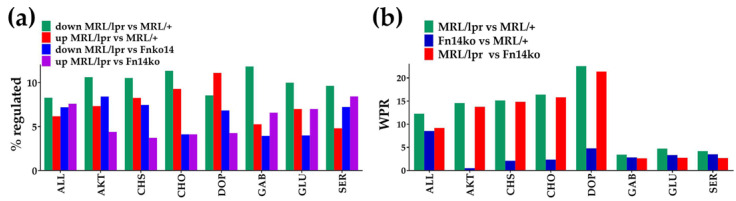
Overall transcriptomic differences in the analyzed pathways among the three phenotypes. (**a**) Percentages of up- and down-regulated genes in selected pathways for indicated comparisons. (**b**) Weighted Pathway Regulation (WPR) of selected pathways. AKT, PI3K-AKT pathway; CHS, chemokine signaling pathway; CHO, cholinergic neurotransmission; DOP, dopaminergic neurotransmission; GAB, GABAergic neurotransmission, GLU, glutamatergic neurotransmission; SER, serotonergic neurotransmission.

Figure 3a presents the percentages of up- and down-regulated genes within several relevant pathways, including PI3K-AKT (AKT), chemokine-signaling (CHS), and neurotransmission pathways. Figure 3b illustrates the weighted pathway regulation (WPR) scores of these pathways when comparing the three phenotypes. Notably, while there are no distinguishable differences between the models in the GABAergic (GAB), glutamatergic (GLU), and serotonergic (SER) pathways; the PI3K-AKT and chemokine-signaling, as well as the cholinergic (CHO) and dopaminergic (DOP) neurotransmission pathways are differentially regulated in the MRL/lpr mice compared with Fn14ko and MRL/+ controls. The low WPRs, when the 2 controls are compared with each other (Figure 3b; blue bars), indicate similar overall regulation of these pathways in the 2 control phenotypes; thus, pointing to a TWEAK/Fn14-mediated effect on those pathways that are differentially regulated.

### 3.4. Gene Hierarchy in the Hippocampus

The more important a gene is in preserving a particular phenotype, the more protected are its sequence and expression level by the cellular homeostatic mechanisms. In addition, critical genes play an important role in the regulation of major functional pathways, which can be evaluated through analyzing their expression coordination with the pathways’ genes. Combining the measure of expression control and expression coordination with other genes in the phenotype provides the Gene Commanding Height (GCH) score. We used GCH analysis as detailed in [20,31,32,33] to establish the gene hierarchy in the hippocampi of the three mouse models. Table 3 presents the top 15 genes with the highest GCH in each phenotype. 

Of particular interest are the top GCH genes in the MRL/+ as these are important in the preservation of the healthy, non-lupus phenotype in this background control model. As expected, most of the genes encode for household proteins required for genetic material transcription and protection/repair regulation, intracellular structural and transportation mechanisms, and modulation of cell differentiation, proliferation, and signal transduction. Notably, one of the top GCH genes, Synaptotagmin XI (*Syt11*), plays an important role in regulating endocytosis and the vesicle-recycling process identified to be particularly significant in dopamine transmission, in addition to inhibiting cytokine secretion, such as interleukin-6 (IL6) and tumor necrosis factor (TNF), in macrophages and microglia [44]—both of these functions are potentially important in preventing the aberrant processes occurring in the lupus-prone brain.

The lack in overlap of the top GCH genes between the 3 phenotype is apparent. This is especially noteworthy when comparing the MRL/lpr model with the Fn14ko. As we discussed in our recent publication [20], while Fn14ko is thought to be physiologically similar to the MRL/lpr except for the knockout of 1 gene, transcriptomically, it appears that the differences between the models are more extensive and affect many more genes (in addition to the knocked down tweak) and functional pathways than would be expected.

### 3.5. Phenotype Dependence of the PI3K-AKT Pathway 

**Figure 4:**
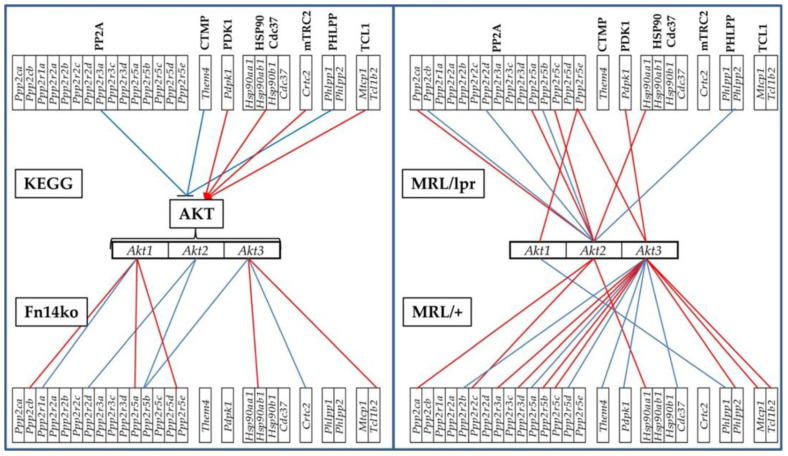
Phenotype-dependent transcriptomic network of AKT genes with their KEGG-derived activator and inhibitor genes in the hippocampus. Red/blue lines depict statistically significant (*p* < 0.05) expression synergism/antagonism between the linked genes. Red line indicates synergism, and blue line antagonism between the 2 genes.

**Figure 5:**
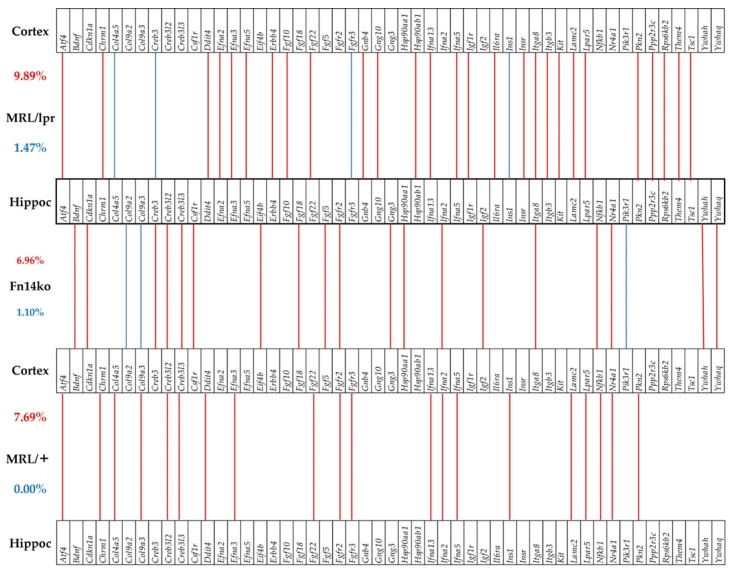
Significant in-phase and anti-phase expression of PI3K-AKT genes between the cortex and hippocampus in each phenotype. Red/blue lines indicate the genes that are significantly expressed in-phase and anti-phase between the two regions. Red numbers indicate percentages of in-phase expression, and blue numbers indicate percentages of anti-phase expression.

The correlation expression levels of each of the AKT genes with their KEGG-established stimulators and inhibitors were analyzed (using Equation (3) in Materials and Methods section). Statistically significant (*p* < 0.05) COR of the AKT genes with their KEGG-determined stimulators and inhibitors [45] in the hippocampi of the three phenotypes are shown in Figure 4. According to the established and widely-used KEGG, the genes included in the groups labeled as PDK1, HSP90/Cd37, mTRC2, and TCL1 should be synergistically expressed (red lines) with all genes from the AKT group as they are thought to stimulate *Akt* gene expression within the pathway, while the genes labeled PP2A, CTMP and PHLPP should be antagonistically expressed (blue lines) with the *Akt* genes. As revealed by the coordination analysis in Figure 4, the genes are not uniformly correlated among the phenotypes, or consistently in-line with the KEGG-determined expected associations. The expression coordination is strongly dependent on the phenotype, contrary to the claimed universality of the KEGG-determined pathway. Interestingly, there are also substantial differences with expression coordination of the same genes between the hippocampus and cortex of the same phenotypes (Figure 7 in [20], as well as Figure 5 below).

### 3.6. Phenotype-Dependent Cortex-Hippocampus Synchronous Expression of Genes 

**Figure 6:**
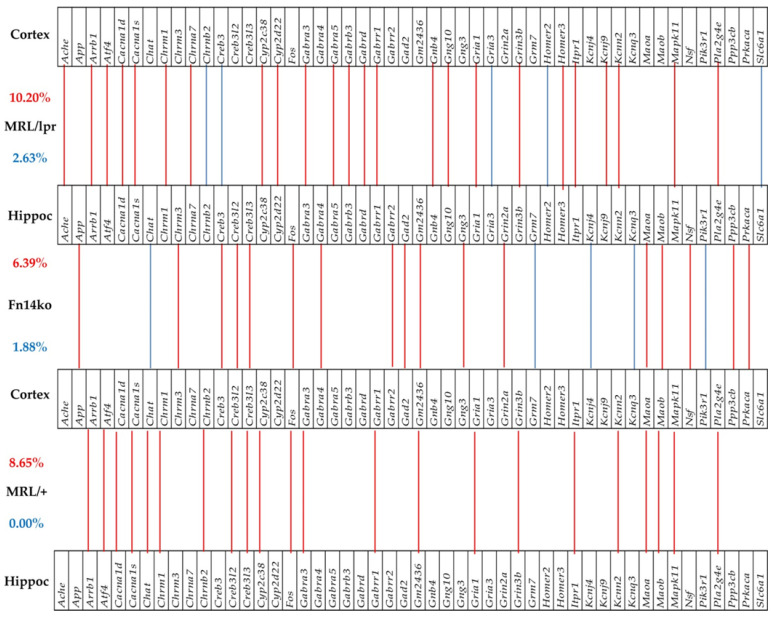
Significant in-phase and anti-phase expression of neurotransmission genes between the cortex and hippocampus in each phenotype. Red/blue lines indicate the genes that are significantly expressed in-phase and antiphase between the two regions. Red numbers indicate percentages of in-phase, and blue numbers of anti-phase expressions.

Cognitive dysfunction in NPSLE has been frequently associated with hippocampal functional changes [21,22,23,24,25,26]. Previously, we presented data of gene expression analysis in the cortex of MRL/lpr, Fn14ko, and MRL/+ mice [20]. Figure 5 and Figure 6 illustrate the correlation between gene expression in the cortex, compared with the hippocampus of the 3 phenotypes, in the PI3K/AKT and neurotransmission pathways, respectively. These figures focus on the genes that are significantly expressed in-phase (expression levels are in the same direction, either enhanced or suppressed) and antiphase (gene expression is in opposite directions) among the two brain regions of each phenotype. As shown, there is enhanced in-phase gene expression between the regions in the MRL/lpr phenotype in about 10% of the analyzed genes in both pathways. However, the pattern of this association, both in the extent of general in-phase expression and which genes are synchronously expressed is phenotype-specific. Furthermore, when focusing on the asynchronously expressed genes between the 2 regions (blue lines), it is interesting that the lupus-prone MRL/lpr mice show the most asynchronous expression of genes in both analyzed pathways, while the MRL/+ controls show none.

## 4. Discussion

Analysis of the differential genomic expression and regulation of the hippocampi of lupus-prone mice highlights several important pathways that may play a role in inducing the NPSLE phenotype. More specifically, we focused on those pathways that appear to be TWEAK/Fn14-dependent, as TWEAK is an established key player in the pathogenesis of NPSLE [14,15,17,46], and an improved understanding of its downstream effects can provide important insight into the underlying pathologic processes, including potential targets for intervention. Our analysis highlights the importance of *Akt2* in particular, and the PI3K-AKT pathway in general, in the genomic regulation of the MRL/lpr lupus-prone mouse. In addition to the PI3K-AKT pathway, other significant hippocampal pathways that seem to be associated with TWEAK/Fn14 activation are chemokine signaling, cholinergic, and dopaminergic neurotransmission pathways. In addition to these highlighted pathways, we also present genes that have significant impact on the MRL/lpr hippocampus transcriptome, such as *Dnajc28*, *Syne2*, and suppressed levels of *Ttr*, among others. Finally, we present evidence to differential pathway progression, or alterations to expected pathway sequences, as predicted by KEGG between the different mice phenotypes.

The hippocampus has long been implicated as a focus of NPSLE memory and learning impairment. In studies using advanced imaging techniques, the hippocampus is among the most consistently affected brain regions [3,47]. The specific localization of pathology to certain areas of the brain can be related to mechanical differences between the regions, such as increased regional vulnerability of the blood-brain barrier (BBB) increasing the local influx of inflammatory factors [48], as well as differences in local cell populations potentially making them selectively vulnerable to the NPSLE inflammatory drivers [49]. In addition, it has been demonstrated that different brain regions have variable cytokine profiles in the setting of NPSLE [50,51]. It was, therefore, important for us to specifically examine gene expression changes in the hippocampus, as it is likely more relevant to NPSLE neurocognitive changes than an evaluation that is not region-specific.

The neuropsychiatric manifestations of SLE have been shown to be triggered by an inflammatory process in a variety of contexts. Blood-brain-barrier (BBB) disruption allows infiltration of pathogenic antibodies to brains of mouse models, thereby causing neuropsychiatric manifestations [52]; tertiary lymph nodes at the site of the choroid plexus enable activated T- and B-cells to migrate to the brain parenchyma [53]; activated microglia cells are thought to play an active role in the local inflammatory process in this setting; abundance of pro-inflammatory cytokines is found in the CSF of NPSLE patients (reviewed in [1]). It is, therefore, not surprising that many of the NPSLE symptoms manifest in the setting of active SLE disease, and improve with immunosuppression. However, neurocognitive dysfunction in lupus patients has been a more elusive, less overtly inflammation-driven process. In human disease, it can often appear when overall disease activity is quiescent, and the symptoms often do not respond to immunosuppression. Previously, our group has shown in the MRL/lpr model that even with drastic attenuation of systemic inflammation, the neurocognitive behavioral phenotype persists, along with local cytokine production and neurodegeneration [54,55]. Accordingly, many of the most differentially regulated genes in the hippocampus of the lupus mouse model are ones that are related to neurodegenerative conditions, such as Alzheimer’s and Parkinson’s Disease, more than typical autoimmune, inflammation-related genes. At the same time, the prognosis of Alzheimer’s patients is associated with degree of systemic and local inflammation [56], and inflammatory processes, such as local cytokine production and T-cell infiltration, have been shown to play an important role in neurodegenerative conditions [57,58]. Thus, the interplay between inflammation and neurodegeneration is an important one to further explore.

*Dnajc28* is a member of the Heat Shock Protein 40 (Hsp40) family. The members of this family of HSPs are thought to be molecular co-chaperones that bind to Hsp70 members, allowing them to interact with client proteins facilitating their proper folding, intracellular trafficking, and marking specific proteins for degradation [35]. A number of the Hsp40 family members have been implicated in familial forms of Parkinson’s Disease [35]. At the same time, several studies have shown neuroprotective effects of increased levels of extracellular HSPs, including Hsp40 and Hsp70, in several neurodegenerative disease models [59,60,61,62]. Thus, it is yet unclear whether the excess expression of *Dnajc28* in the MRL/lpr model is damaging in and of itself, or whether its overexpression is a compensatory response to the stressed local environment. Similarly, *Syne2* (spectrin repeat containing nuclear envelope protein 2), a member of the LINC (Linker of Nucleoskeleton and Cytoskeleton) complex that tethers the nuclear lamina to the cytoskeleton [63], has been previously identified to be associated with Alzheimer’s Disease (AD) and familial early-onset dementia [38,39]. *Ttr* is a systemic amyloid precursor that with abnormal folding due to genetic mutations or aging can lead to a form of systemic amyloidosis. In the setting of AD, it seems to have a protective effect by binding with the Aβ amyloid peptides and preventing fibril formation [42,64,65]. Interestingly, while historically Ttr was thought to be produced only by choroid plexus epithelial cells in the CNS, several groups have shown neuronal production of Ttr, particularly in the hippocampus and cortex, likely induced by Aβ precursor peptides as a local protective mechanism [42]. In our study, the increased expression of *Ttr* in the hippocampus of the MRL/lpr lupus model, compared with both the MRL/+ and Fn14ko controls, may indicate a compensatory, protective mechanism that was driven in the lupus mice due to stress or local inflammation (there is no evidence in the literature for increased production of amyloid precursor proteins in the context of SLE, or NPSLE [66], and so it is unclear what is driving the increased expression of *Ttr* here).

Similar to our previous findings in the MRL/lpr cortex, Akt2 appears to play a central role in the TWEAK/Fn14-induced effects of the hippocampus of the lupus model. Akt2 is one of 3 closely related serine/threonine-protein kinases (Akt1-3) that are key members of the PI3K-AKT pathway; regulating many essential processes, including cell proliferation and survival, growth, metabolism, and angiogenesis [67]. Studies of specific *Akt1-3* null mice provided information regarding differences in roles and functions of the 3 isoforms. Akt3 is the most abundant in the brain, and plays a role in brain development and neurodegeneration [68]. *Akt3*-null mice have 25% smaller brain size [69], and increased susceptibility to demyelination in experimental autoimmune encephalitis (EAE), a widely used model for multiple sclerosis. Akt1 overexpression promotes enhanced myelination [70]. Conversely, *Akt2^-/-^* mice, lacking the Akt2 isoform that is known to be the most crucial in insulin-mediated glucose regulation [71], have normal brain size [69]. This lack of obvious brain manifestations in *Akt2*-null mice make our findings more curious. We show here that *Akt2* is of the most differentially expressed genes in the hippocampus of MRL/lpr with significant WIR, indicating substantial effect on the MRL/lpr transcriptome and presumably phenotype. As *Akt2* expression remains comparable to background control in the Fn14ko mice, the *Akt2* overexpression in the MRL/lpr mice is likely mediated by TWEAK/Fn14 activation. TWEAK-Akt association has been shown in other systems, such as the heart [72], skeletal muscles [73], and tumors [74,75], including glioma, where Akt2 was specifically implicated in mediating TWEAK-induced cell survival [76]. Thus, it is conceivable that, while Akt2 does not play a major ongoing role in brain development and function, it can be activated in CNS inflammatory conditions such as NPSLE by TWEAK activity, possibly as a compensatory mechanism to improve cell survival in the context of inflammation-induced damage. *Akt2^-/-^* mice can be used to further explore whether Akt2 plays a direct pathogenic role contributing to the NPSLE-like phenotype, thereby clarifying whether Akt2 inhibition can be a viable and effective treatment in such scenarios.

In addition to the PI3K-AKT pathway, the chemokine-signaling pathway, as well as the cholinergic and dopaminergic neurotransmission pathways, were identified as important within the TWEAK/Fn14-mediated processes in the lupus-model hippocampus. Chemokine-signaling is an expected downstream event, especially in a cytokine-induced inflammatory context, as is the case in SLE in general, and in our experimental setting that focused on downstream effects of TWEAK/Fn14 activation. Highlighting the role of the cholinergic and dopaminergic neurotransmission pathways, as opposed to others, as significant in the pathogenesis of NPSLE is an important step in elucidating contributing mechanisms, as well as identifying targets of intervention for prevention and treatment.

Importantly, we also demonstrate that our current tools for genetic and pathway evaluations require further refinement. For example, KEGG-determined pathways widely relied upon for pathway identification and prediction studies, seem to be phenotype-dependent, and not universal across mice models. Furthermore, as we previously discussed in [20], gene knockout models, used in studies to isolate effects of one particular gene, display much more pervasive genetic and phenotypic differences from their controls. These observations should be taken into account when making predictions and trying to reach conclusions based on these methods. Of course, this presents an important limitation to our own study, and it should, therefore, be emphasized that this is an exploratory evaluation, and further confirmatory investigations need to be pursued prior to making definitive conclusions. These future studies should include protein analysis beyond gene expression, as there can be significant differences between a gene expression level and its actual translation. Another important limitation of our study is that we utilized only one mouse model: the MRL/lpr and its background control. Further studies need to be undertaken in other mice models to confirm our findings as relevant in NPSLE in general, and not just within the context of the MRL/lpr model.

## 5. Conclusions

SLE is a systemic autoimmune disease that drives inflammation in a myriad of organs, including the brain. While inflammation is a critical piece of the puzzle and probably its initial trigger, it is likely not the only driver of neuropsychiatric symptoms. Clinically, common and prevalent neuropsychiatric manifestations of SLE, such as cognitive dysfunction and mood disorders, often occur independent of disease activity (thus, not during times of increased systemic inflammation) and do not respond to immunosuppression. In this study, we focused on the effects of the pro-inflammatory cytokine TWEAK and its cognate receptor, Fn14, known to play an important role in NPSLE, on the transcriptome of the hippocampus of a lupus-prone mouse model. Notably, many of the most differentially regulated genes and pathways identified are those involved in neurodegenerative processes, as opposed to inflammatory ones, including *Dnajc28*, *Ttr*, and the PI3K-AKT pathway. Transitioning the focus of study to relevant neurodegenerative mechanisms, that potentially contribute to neuropsychiatric manifestations of SLE, may provide a clearer understanding of the underlying pathophysiology and enable the identification of effective treatment modalities that have, so far, remained elusive.

## Figures and Tables

**Table 1:** The 20 most highly expressed genes in the hippocampus of MRL/lpr mice. Average values of gene expression (AVE) are expressed in the table as multiples of the median gene expression.

Gene	Description	MRL/lpr	MRL/+	Fn14ko
*Dnajc28*	DnaJ (Hsp40) homolog, subfamily C, member 28	334.06	0.17	0.09
*Syne2*	synaptic nuclear envelope 2	316.02	0.18	0.09
*Rpp38*	ribonuclease P/MRP 38 subunit (human)	314.63	1.03	1.31
*Cox7b*	cytochrome c oxidase subunit VIIb	311.86	23.40	31.39
*Kctd10*	potassium channel tetramerisation domain containing 10	307.63	0.32	0.09
*Tle6*	transducin-like enhancer of split 6, homolog of Drosophila E	307.41	0.37	0.54
*Pcnx4*	pecanex homolog 4	306.69	1.26	1.81
*Dlx5*	distal-less homeobox 5	302.19	0.48	0.14
*Clec14a*	C-type lectin domain family 14, member a	300.99	0.21	0.14
*Coq6*	coenzyme Q6 homolog (yeast)	290.92	2.95	3.18
*Cradd*	CASP2 and RIPK1 domain containing adaptor with death domain	289.92	0.40	0.60
*Ppp2ca*	protein phosphatase 2 (formerly 2A), catalytic subunit, α isoform	289.29	12.08	19.16
*Gps1*	G protein pathway suppressor 1	282.09	19.02	21.20
*Zfp455*	zinc finger protein 455	276.70	0.20	0.12
*Donson*	downstream neighbor of SON	267.23	0.83	1.36
*Tmie*	transmembrane inner ear	265.98	0.43	0.32
*Akt2*	thymoma viral proto-oncogene 2	261.94	4.09	7.64
*Deaf1*	deformed epidermal autoregulatory factor 1 (Drosophila)	259.89	1.35	0.82
*Eif2s1*	eukaryotic translation initiation factor 2, subunit 1 α	258.35	0.55	0.84
*Dnajc14*	DnaJ (Hsp40) homolog, subfamily C, member 14	256.52	0.77	0.63

MRL/MpJ-Fas^lpr/lpr^ lupus model (MRL/lpr); MRL/MpJ background control (MRL/+); MRL/lpr Fn14 knock-out (Fn14ko)

**Table 2:** The 60 genes that are the largest contributors to the MRL/lpr phenotype. Positive values indicate upregulation of the gene in the MRL/lpr compared with MRL/+ and Fn14ko, and negative values indicate its downregulation. Shaded genes are ones with a similar trend in both control phenotypes, indicating that TNF-like weak inducer of apoptosis (TWEAK)/Fn14 activation plays a role in the gene’s regulation. “x”, expression ratio/fold-change; “WIR”, Weighted Individual (gene) Regulation.

Gene	Description	MRL/lpr vs. MRL/+		MRL/lpr vs. Fn14ko	
		x	WIR	x	WIR
*Nrtn*	neurturin	−17.12	−1032.44	−1.11	−0.24
*Dnajc28*	DnaJ (Hsp40) homolog, subfamily C, member 28	1979.55	203.31	3817.12	203.39
*Syne2*	synaptic nuclear envelope 2	1714.27	192.30	3672.93	192.40
*Rpp38*	ribonuclease P/MRP 38 subunit (human)	304.48	190.96	241.04	190.68
*Tle6*	transducin-like enhancer of split 6, homolog of Drosophila E(spl)	830.65	187.22	572.06	187.05
*Kctd10*	potassium channel tetramerisation domain containing 10	950.07	187.04	3585.62	187.28
*Dlx5*	distal-less homeobox 5	635.75	183.64	2170.24	183.99
*Clec14a*	C-type lectin domain family 14, member a	1421.76	183.14	2104.76	183.21
*Cox7b*	cytochrome c oxidase subunit VIIb	13.33	178.48	9.93	170.27
*Cradd*	CASP2 and RIPK1 domain containing adaptor with death domain	719.92	176.35	484.24	176.15
*Coq6*	coenzyme Q6 homolog (yeast)	98.49	175.50	91.40	175.27
*Ppp2ca*	protein phosphatase 2 (formerly 2A), catalytic subunit, α isoform	23.95	169.92	15.10	162.69
*Zfp455*	zinc finger protein 455	1373.60	168.35	2255.80	168.43
*Donson*	downstream neighbor of SON	322.96	162.56	196.84	162.02
*Tmie*	transmembrane inner ear	618.70	161.70	818.52	161.81
*Gps1*	G protein pathway suppressor 1	14.83	160.90	13.31	158.67
*Akt2*	thymoma viral proto-oncogene 2	64.02	158.94	34.28	155.29
*Deaf1*	deformed epidermal autoregulatory factor 1 (Drosophila)	193.09	157.27	316.93	157.80
*Eif2s1*	eukaryotic translation initiation factor 2, subunit 1 α	472.78	157.09	307.81	156.79
*Dnajc14*	DnaJ (Hsp40) homolog, subfamily C, member 14	332.10	155.60	410.38	155.75
*Arrdc1*	arrestin domain containing 1	461.59	155.23	406.91	155.15
*Nkap*	NFKB activating protein	−23.74	−146.42	1.12	0.04
*Sall1*	sal-like 1 (Drosophila)	515.64	142.36	977.24	142.58
*Rnmtl1*	RNA methyltransferase like 1	286.57	141.15	314.45	141.22
*Ybx2*	Y box protein 2	799.94	139.73	707.63	139.70
*Arl4a*	ADP-ribosylation factor-like 4A	203.58	139.32	117.37	138.48
*Tbc1d19*	TBC1 domain family, member 19 (Tbc1d19), mRNA [NM_144517]	259.29	136.98	213.37	136.79
*Lrrc2*	leucine rich repeat containing 2	1145.66	135.04	1901.54	135.11
*Gpr183*	G protein-coupled receptor 183	1187.49	131.43	2459.92	131.53
*Abcc3*	ATP-binding cassette, sub-family C (CFTR/MRP), member 3	988.46	131.05	1673.97	131.14
*Ccdc124*	coiled-coil domain containing 124	16.46	121.35	13.70	118.72
*Rgl1*	ral guanine nucleotide dissociation stimulator-like 1	113.72	118.88	182.93	119.55
*Mki67*	antigen identified by monoclonal antibody Ki 67	903.22	101.46	977.16	101.48
*Hba-a1*	hemoglobin α, adult chain 1	3.69	91.05	1.82	54.31
*Hba-a2*	hemoglobin α, adult chain 2	2.54	90.32	1.77	64.14
*Tank*	TRAF family member-associated Nf-kappa B activator	381.55	87.10	407.55	87.12
*Slc10a1*	solute carrier family 10 (sodium/bile acid cotransporter family), member 1	1.82	83.41	1.82	83.18
*H2-Gs10*	MHC class I like protein GS10	10.38	82.27	9.02	80.23
*Rnf168*	ring finger protein 168	87.22	80.35	106.75	80.63
*Elk1*	ELK1, member of ETS oncogene family	1.59	65.37	1.75	75.96
*Exosc2*	exosome component 2	1.74	62.55	1.39	39.39
*Cxcl11*	chemokine (C-X-C motif) ligand 11	2.82	61.18	2.68	59.47
*Ttr*	transthyretin	−2.00	−52.38	−1.76	−40.27
*Kif5a*	kinesin family member 5A	1.69	52.29	1.30	26.92
*Hccs*	holocytochrome c synthetase	3.37	50.04	3.14	48.37
*Zfp628*	zinc finger protein 628	−1.34	−47.43	1.36	28.96
*Atp6v0c*	ATPase, H+ transporting, lysosomal V0 subunit C	−1.40	−43.10	−1.18	−11.73
*Gla*	galactosidase, α	2.04	41.87	1.61	31.24
*Psap*	prosaposin	−1.66	−41.72	−1.16	−7.03
*Dcc*	deleted in colorectal carcinoma	2.30	41.29	3.40	52.26
*Oxt*	oxytocin	−16.11	−39.14	1.02	0.00
*Tubb2a*	tubulin, β 2A	−1.35	−38.27	−1.41	−36.56
*Ddn*	dendrin	−2.34	−38.13	−1.28	−3.17
*Ptms*	parathymosin	−1.44	−37.91	1.19	8.51
*Acot7*	acyl-CoA thioesterase 7	−1.71	−35.06	−1.15	−3.35
*Ptgds*	prostaglandin D2 synthase (brain)	1.45	34.82	1.23	11.03
*Thy1*	thymus cell antigen 1, theta	−1.32	−34.56	1.18	11.17
*Sparcl1*	SPARC-like 1	1.42	33.77	1.01	0.13
*Pkm2*	pyruvate kinase, muscle	−1.57	−33.58	−1.13	−3.69
*Cox8a*	cytochrome c oxidase, subunit VIIIa	1.31	32.71	1.19	17.94

**Table 3:** The top 15 genes in each phenotype. The numbers are the gene commanding heights (GCH) in the indicated phenotypes. Top genes have the highest GCHs.

Gene	Description	MRL/+	MRL/lpr	Fn14ko
*Tfb2m*	transcription factor B2, mitochondrial	234	3	9
*Hiatl1*	hippocampus abundant transcript-like 1	85	4	2
*Mettl11a*	methyltransferase like 11A	73	1	4
*Gipc1*	GIPC PDZ domain containing family, member 1	68	2	1
*Homez*	homeodomain leucine zipper-encoding gene	60	2	2
*Syt11*	synaptotagmin XI	58	2	3
*Eef1g*	eukaryotic translation elongation factor 1 γ	54	4	2
*Brsk1*	BR serine/threonine kinase 1	53	2	2
*Gga2*	golgi associated, γ adaptin ear containing, ARF binding protein 2	50	1	2
*St3gal5*	ST3 β-galactoside α-2,3-sialyltransferase 5	49	1	2
*Dusp22*	dual specificity phosphatase 22	46	2	1
*Avl9*	AVL9 homolog	42	2	3
*Foxq1*	forkhead box Q1	42	1	1
*Lmna*	lamin A	42	3	1
*Anpep*	alanyl (membrane) aminopeptidase	41	1	1
*Xrcc4*	X-ray repair complementing defective repair in Chinese hamster cells 4	4	43	1
*Tubb4*	tubulin, β 4	2	43	2
*Arhgap23*	Rho GTPase activating protein 23	2	36	3
*Ecd*	ecdysoneless homolog	5	35	5
*Cacfd1*	calcium channel flower domain containing 1	5	29	2
*Nacad*	NAC α domain containing	10	26	2
*Pnldc1*	poly(A)-specific ribonuclease (PARN)-like domain containing 1	3	23	2
*Mrpl40*	mitochondrial ribosomal protein L40	1	22	3
*Serf2*	small EDRK-rich factor 2	6	22	2
*Rpa2*	replication protein A2	7	22	3
*Unc79*	unc-79 homolog	4	20	5
*Pogk*	pogo transposable element with KRAB domain	8	19	3
*Gabpb1*	GA repeat binding protein, β 1	2	18	5
*Mad2l2*	MAD2 mitotic arrest deficient-like 2	3	18	10
*Ccdc101*	coiled-coil domain containing 101	2	18	2
*Card19*	caspase recruitment domain family, member 19	2	2	27
*Ppfia1*	protein tyrosine phosphatase, receptor type, f polypeptide, interacting protein (liprin), α 1	6	5	26
*Map4k5*	mitogen-activated protein kinase kinase kinase kinase 5	2	2	25
*Ddx59*	DEAD (Asp-Glu-Ala-Asp) box polypeptide 59	2	1	25
*Fbxo25*	F-box protein 25	2	1	21
*Rg9mtd3*	RNA (guanine-9-) methyltransferase domain containing 3	3	2	20
*Iscu*	IscU iron-sulfur cluster scaffold homolog, nuclear gene encoding mitochondrial protein	3	3	20
*Gm14407*	60S ribosomal protein L27a pseudogene	3	2	19
*Riok2*	RIO kinase 2	3	5	19
*Slc25a47*	solute carrier family 25, member 47	3	2	19
*Mapre3*	microtubule-associated protein, RP/EB family, member 3	9	2	19
*Pomc*	pro-opiomelanocortin-α	3	5	18
*Polr1e*	polymerase (RNA) I polypeptide E	6	3	18
*Olfr1347*	olfactory receptor 1347	3	4	17
*Tor2a*	torsin family 2, member A	9	1	16

## Data Availability

Raw and normalized gene expression data were deposited and are publicly accessible at https://www.ncbi.nlm.nih.gov/geo/, (accessed on 25 March 2021) as GSE164140 (cortex) and GSE169486 (hippocampus).

## Supplementary Materials

The following are available online at https://www.mdpi.com/article/10.3390/genes12081172/s1, Table S1: The 20 most stably expressed genes (low relative expression variability, REV) in each phenotype; Table S2: The 20 most unstably expressed genes (high REV) in each phenotype.
