# Supplementary material for: TWEAKing the Hippocampus: The Effects of TWEAK on the Genomic Fabric of the Hippocampus in a Neuropsychiatric Lupus Mouse Model
Source: Genes (Basel). 2021 Jul 29;12(8):1172. doi: 10.3390/genes12081172 (PMC8392718; doi:10.3390/genes12081172)
Supplement: Supplementary file 1 [file genes-12-01172-s001.zip › genes-1292935-supplementary.pdf]

genes MDPI

Supplementary Materials

Table S1. The 20 most stably expressed genes (low relative expression variability, REV) in each of the three phenotypes. Shaded genes are the genes of interest in each phenotype.

Gene	Description	MRL/*lpr*	Fn14ko	MRL/+
Xrcc4	X-ray repair complementing defective repair in Chinese hamster cells 4	0.66	18.31	12.04
Tubb4	tubulin, beta 4	0.73	15.07	18.02
Ecd	ecdysoneless homolog (Drosophila)	1.27	4.64	23.95
Arhgap23	Rho GTPase activating protein 23	1.28	7.90	16.16
Ggnbp2	gametogenetin binding protein 2	1.36	10.69	10.32
Tusc2	tumor suppressor candidate 2	1.40	17.41	15.19
Med22	mediator complex subunit 22	1.42	8.15	20.64
Mfsd5	major facilitator superfamily domain containing 5	1.42	9.11	8.62
Acbd3	acyl-Coenzyme A binding domain containing 3	1.49	14.69	13.51
Nacad	NAC alpha domain containing	1.51	15.95	9.89
Pdia3	protein disulfide isomerase associated 3	1.52	15.96	22.67
Serf2	small EDRK-rich factor 2	1.53	10.49	21.71
Hax1	HCLS1 associated X-1	1.55	16.20	16.17
Pnldc1	poly(A)-specific ribonuclease (PARN)-like domain containing 1	1.56	11.55	44.31
Cacfd1	calcium channel flower domain containing 1	1.77	16.68	25.14
Mad2l2	MAD2 mitotic arrest deficient-like 2	1.78	2.31	11.33
Ppp1r12c	protein phosphatase 1, regulatory (inhibitor) subunit 12C	1.82	11.85	16.76
Rpa2	replication protein A2	1.88	8.77	11.51
Unc79	unc-79 homolog (C. elegans)	1.89	4.81	32.56
Nfasc	neurofascin	1.90	17.03	24.38
Ddx59	DEAD (Asp-Glu-Ala-Asp) box polypeptide 59	58.12	0.97	21.59
Rg9mtd3	RNA (guanine-9-) methyltransferase domain containing 3	6.77	0.98	47.38
Ppfia1	protein tyrosine phosphatase, receptor type, f polypeptide (PTPRF), interacting protein (liprin), alpha 1	9.43	1.09	6.35
Cenpv	centromere protein V	8.75	1.14	33.88
Polr1e	polymerase (RNA) I polypeptide E	7.16	1.17	20.01
Pclo	piccolo (presynaptic cytomatrix protein)	31.49	1.17	19.95
Map4k5	mitogen-activated protein kinase kinase kinase kinase 5	15.50	1.18	19.31
Mrpl45	mitochondrial ribosomal protein L45	9.22	1.24	22.10
Iscu	IscU iron-sulfur cluster scaffold homolog (E. coli) (Iscu), nuclear gene encoding mitochondrial protein	8.15	1.31	8.60
Riok2	RIO kinase 2 (yeast)	5.43	1.37	22.03
Nup88	nucleoporin 88	13.52	1.39	26.28
Fbxo25	F-box protein 25	40.03	1.44	79.33
Plcb1	phospholipase C, beta 1	13.38	1.44	61.35
Slc6a17	solute carrier family 6 (neurotransmitter transporter), member 17	10.46	1.53	10.30
Pomc	pro-opiomelanocortin-alpha	4.61	1.53	35.08
Tor2a	torsin family 2, member A	45.22	1.55	13.89
Ccdc134	coiled-coil domain containing 134	7.10	1.60	16.73
Gpr137b-ps	G protein-coupled receptor 137B, pseudogene	9.18	1.61	14.52
Pdcd2	programmed cell death 2	10.57	1.62	24.11
Gm14407	predicted gene 14407	18.82	1.71	41.59
Tfb2m	transcription factor B2, mitochondrial	15.69	2.65	0.28
Homez	homeodomain leucine zipper-encoding gene	23.29	15.77	0.94
Ttc26	tetratricopeptide repeat domain 26	19.30	15.56	0.98

Hiatl1	hippocampus abundant transcript-like 1	13.97	9.72	1.36
Galns	galactosamine (N-acetyl)-6-sulfate sulfatase	25.09	21.35	1.38
Dfna5	deafness, autosomal dominant 5 (human)	20.16	10.87	1.45
Avl9	AVL9 homolog (S. cerevisiase)	14.70	5.70	1.48
Dele1	DAP3 binding cell death enhancer 1	18.28	11.55	1.55
Foxq1	forkhead box Q1	19.66	26.03	1.58
Gipc1	GIPC PDZ domain containing family, member 1	20.12	24.64	1.68
Nfkbib	nuclear factor of kappa light polypeptide gene enhancer in B-cells inhibitor, beta	12.98	9.89	1.70
Tor1aip2	torsin A interacting protein 2	39.31	12.54	1.72
Abhd8	abhydrolase domain containing 8	28.32	20.92	1.74
Ndst2	N-deacetylase/N-sulfotransferase (heparan glucosaminyl) 2	8.64	9.60	1.79
Trim37	tripartite motif-containing 37	41.95	12.85	1.86
Cnnm4	cyclin M4	16.35	4.75	1.90
Lmna	lamin A	15.22	16.69	1.92
Gga2	golgi associated, gamma adaptin ear containing, ARF binding protein 2	19.14	17.78	1.97
Mettl11a	methyltransferase like 11A	39.50	6.43	1.99
Syt11	synaptotagmin XI	13.12	9.44	2.07

Table S2. The 20 most unstably expressed genes (high REV) in each of the three phenotypes. Shaded genes are the genes of interest in each phenotype.

Gene	Description	MRL/lpr	Fn14ko	MRL/+
Kctd10	potassium channel tetramerisation domain containing 10	200.0	43.4	97.5
Syne2	synaptic nuclear envelope 2	200.0	25.3	73.7
Vprbp	Vpr (HIV-1) binding protein	199.9	29.0	75.6
Dnajc28	DnaJ (Hsp40) homolog, subfamily C, member 28	199.9	10.8	49.4
Gpr183	G protein-coupled receptor 183	199.9	18.9	70.8
Zfp455	zinc finger protein 455	199.9	11.0	37.4
Clec14a	C-type lectin domain family 14, member a	199.9	21.7	39.5
Lrrc2	leucine rich repeat containing 2	199.9	5.5	55.8
Abcc3	ATP-binding cassette, sub-family C (CFTR/MRP), member 3	199.9	36.0	41.2
Mki67	antigen identified by monoclonal antibody Ki 67	199.9	29.9	55.1
Dlx5	distal-less homeobox 5	199.8	21.5	48.8
Tmie	transmembrane inner ear	199.7	15.7	53.2
Sall1	sal-like 1 (Drosophila)	199.7	5.5	37.7
Ybx2	Y box protein 2	199.7	25.9	27.0
Dnajc14	DnaJ (Hsp40) homolog, subfamily C, member 14	199.7	24.3	24.0
Cradd	CASP2 and RIPK1 domain containing adaptor with death domain	199.7	16.1	27.0
Tank	TRAF family member-associated Nf-kappa B activator	199.5	16.8	6.5
Marf1	meiosis regulator and mRNA stability 1	199.4	24.1	26.1
Eif2s1	eukaryotic translation initiation factor 2, subunit 1 alpha	199.4	15.3	29.1
Tle6	transducin-like enhancer of split 6, homolog of Drosophila E(spl)	199.4	31.7	21.0
Serpina9	serine (or cysteine) peptidase inhibitor, clade A	21.7	159.0	84.8
Gpr88	G-protein coupled receptor 88	85.3	154.6	55.3
Scn4b	sodium channel, type IV, beta	58.2	151.4	12.5
Drd1	dopamine receptor D1	61.8	149.4	58.8
Penk	preproenkephalin	66.9	145.4	66.3
Ppp1r1b	protein phosphatase 1, regulatory (inhibitor) subunit 1B	53.3	134.2	26.5
Adora2a	adenosine A2a receptor	57.3	132.1	87.2
Rgs9	regulator of G-protein signaling 9	21.6	127.2	34.1

Tac1	tachykinin 1	20.5	127.1	38.3
Gpr6	G protein-coupled receptor 6	32.2	116.2	57.4
H2-Ab1	histocompatibility 2, class II antigen A, beta 1	51.2	115.8	62.8
Ccl2	chemokine (C-C motif) ligand 2	52.9	114.5	91.2
Tmem90a	transmembrane protein 90a	21.5	111.9	45.7
Pde10a	phosphodiesterase 10A	39.6	105.1	46.8
Rasd2	RASD family, member 2	47.2	104.2	12.1
Lyz1	lysozyme 1	64.0	103.7	64.0
Rarb	retinoic acid receptor, beta	26.5	102.9	58.7
Pde7b	phosphodiesterase 7B	55.1	101.4	76.0
Lrrc10b	leucine rich repeat containing 10B	93.3	99.5	10.8
Igj	immunoglobulin joining chain	130.0	99.3	106.9
Nkap	NFKB activating protein	3.5	11.6	192.0
Mettl24	methyltransferase like 24	9.9	20.7	164.7
Oxt	oxytocin	31.9	41.0	161.8
Mybpc3	myosin binding protein C, cardiac	56.4	65.0	118.4
Aqp5	aquaporin 5	75.2	73.7	118.1
C7	complement component 7	66.6	68.1	117.9
Olfr681	olfactory receptor 681	62.5	50.2	117.3
Gcnt1	glucosaminyl (N-acetyl) transferase 1, core 2	69.7	65.8	116.6
Ttr	transthyretin	87.8	75.1	116.3
Ifi202b	interferon activated gene 202B	58.4	83.5	115.3
Barx2	BarH-like homeobox 2	84.2	79.9	114.8
Nrtn	neurturin	11.2	29.3	114.5
Zbtb44	zinc finger and BTB domain containing 44	73.2	64.1	114.4
Chrnb1	cholinergic receptor, nicotinic, beta polypeptide 1 (muscle)	51.5	54.5	114.4
Avp	arginine vasopressin	38.7	27.0	114.1
Gm6762	predicted pseudogene 6762	75.8	73.1	113.1
Rab33b	RAB33B, member of RAS oncogene family	52.4	43.6	113.1
Gphb5	glycoprotein hormone beta 5	89.8	75.6	112.4
Tekt2	tektin 2	135.6	52.8	111.6
Ltbp2	latent transforming growth factor beta binding protein 2	52.4	60.9	111.5
